# Dr. John Hauenstein

**Published:** 1905-11

**Authors:** 


Dr. John Hauenstein, of Buffalo, died at his home, 309 Elmwood Avenue, November 10, 1905, aged 84 years. He had been in failing health for about two years, his strong resisting power, founded upon an excellent constitution, maintaining life beyond reasonable expectation.

John Hauenstein was a native of Switzerland and came to this country with his father in 1831. At the age of twelve he became an apprentice in the office of Dr. Dellenbaugh, a circumstance that subsequently led him to take up the study of medicine. His preliminary education was obtained through private teaching and the high school, after which he attended the medical college at Geneva, from which he received his doctorate degree in 1844.

Dr. Hauenstein commenced to practice his profession at once and located his office at Main and Mohawk streets, Buffalo. A few years afterward he removed to 499 Washington street where he maintained his residence and office until 1894, when he retired from active practice after fifty years of arduous professional work. Dr. Hauenstein joined the Medical Society of the County of Erie in 1844, the year of his graduation, became president of the society in 1881, and has read many excellent papers before it. One of the latest of these, entitled, "First Uses of Anesthetics in Buffalo," was read at the seventy-fifth anniversary meeting of the society, January 14, 1896, and was published in this Journal in March of the same year. Another contribution, "A résumé of Fifty Years Obstetric Practice," likewise published in the issue of this JOURNAL for June 1897, was his last medical paper.

Dr. Hauenstein was well known to the medical profession during the fifty years of his active life, and was also one of the distinguished citizens of Buffalo. He was one of the early presidents of the German Young Men's Association and an active member of the Society of Natural Sciences. He was an amiable man, gentle in manner, sweet in disposition and died in the ripeness of years surrounded by a loving, devoted family.

Dr. Hauen stein is survived by his wife, formerly Miss Madeline Sigwalt of this city, whom he married about 60 years ago, and four children, Albert H., Oscar H., Miss Eugenia, and Mrs. Nathaniel Rochester, all of Buffalo.

